# Immunometabolism of Tissue-Resident Macrophages – An Appraisal of the Current Knowledge and Cutting-Edge Methods and Technologies

**DOI:** 10.3389/fimmu.2021.665782

**Published:** 2021-05-07

**Authors:** Giulia Zago, Pedro H. V. Saavedra, Kayvan R. Keshari, Justin S. A. Perry

**Affiliations:** ^1^ Immunology Program, Memorial Sloan Kettering Cancer Center, New York, NY, United States; ^2^ Department of Radiology, Memorial Sloan Kettering Cancer Center, New York, NY, United States; ^3^ Molecular Pharmacology Program, Memorial Sloan Kettering Cancer Center, New York, NY, United States; ^4^ Louis V. Gerstner Jr. Graduate School of Biomedical Sciences, Memorial Sloan Kettering Cancer Center, New York, NY, United States; ^5^ Department of Immunology and Microbial Pathogenesis, Weill Cornell Medical College, New York, NY, United States

**Keywords:** tissue-resident macrophages, metabolism, homeostasis, inflammation, development

## Abstract

Tissue-resident macrophages exist in unique environments, or niches, that inform their identity and function. There is an emerging body of literature suggesting that the qualities of this environment, such as the types of cells and debris they eat, the intercellular interactions they form, and the length of time spent in residence, collectively what we call *habitare*, directly inform their metabolic state. In turn, a tissue-resident macrophage’s metabolic state can inform their function, including whether they resolve inflammation and protect the host from excessive perturbations of homeostasis. In this review, we summarize recent work that seeks to understand the metabolic requirements for tissue-resident macrophage identity and maintenance, for how they respond to inflammatory challenges, and for how they perform homeostatic functions or resolve inflammatory insults. We end with a discussion of the emerging technologies that are enabling, or will enable, *in situ* study of tissue-resident macrophage metabolism.

## Introduction

In his “An Essay Concerning Human Understanding” (1), John Locke famously argued against “innate principles” or the notion that we are born with knowledge that informs our identity. Instead, Locke posited that our identity (and thus uniqueness) arises from sensory experience, “… anything as existing at any determined time and place, we compare it with itself existing at another time, and thereon form the ideas of identity and diversity.” Although Locke’s ideas were ultimately imperfect, his thinking spurred centuries of discussion on the nature of identity: is our identity innately informed or driven by experience? With this as our backdrop, several decades of work on the aptly-named innate immune system has informed a broad understanding of intrinsically-encoded receptors and sensors that serve to fight infection and keep the host healthy. However, with the development of new tools and methods, there is an emerging body of knowledge on how an innate immune cell’s lived experience informs its identity and function. No more so is this true than with the study of tissue-resident macrophages (TRMs).

Specific environmental factors, such as nutrient and oxygen availability, tissue material and debris that TRMs are exposed to, and the length of time spent in this *habitare*, as well as the events that a TRM experience (such as infectious or sterile insult), all factor in to TRM identity and function, an idea that has been beautifully articulated in a series of reviews and prospective articles (2–5). Building from these insights, we propose that each of these factors contribute to the metabolic state of TRMs, which in turn informs the efficiency and nature of TRM function. In this review, we discuss recent advances in our understanding of TRM metabolism, focusing on studies that explore TRM metabolism *in situ*/*in vivo*. We will highlight metabolic regulation of three key aspects of TRM biology: TRM identity, responses during infection and inflammation, and homeostatic/reparative processes. We end with a discussion of methods previously employed to study TRM metabolism as well as emerging technologies that we foresee will lead to a significant advancement in our understanding of TRM metabolism.

## Metabolic Regulation of Tissue-Resident Macrophage Identity

Tissue-resident macrophages (TRMs) are a diverse family of cells that arise from a common erythro-myeloid precursor, develop a core macrophage signature during embryogenesis, and acquire specialized transcriptional programs during organogenesis (6–8). TRMs perform a number of canonical immune functions as well as tissue-specific physiological functions throughout their lifespan (3, 4, 9, 10). For instance, cells in the developing organism begin to undergo apoptosis in the yolk sac which continues in all organs throughout development. These dying cells must be cleared by TRMs to ensure proper organogenesis (11). Osteoclasts, a bone-resident TRM (12), are required for bone resorption and homeostasis, such that perturbed osteoclast activity results in osteopetrosis (diminished osteoclast function) or osteoporosis (excessive osteoclast function) (13). Microglia, a brain-resident TRM, are required for synaptic pruning and optimal neurodevelopment. Alveolar macrophages, a lung-resident TRM, are required for surfactant clearance and airway integrity (4). Adipose tissue TRMs contribute to insulin sensitivity, adipogenesis, and adaptive thermogenesis (14). Bone marrow and splenic red pulp macrophages clear millions of spent erythrocytes each day, essential for maintenance of iron homeostasis and prevention of diseases such as hemochromatosis, thalassemia, and chronic anemia (15). Thus, TRMs are exposed and adapt to a multitude of tissue-specific growth factors (16–19), dying cells and debris with varying metabolic properties (20–27), and unique (and often extreme) tissue environments (3, 28, 29) to ensure tissue and organismal homeostasis.

This is important because TRMs are generally long-lived and self-renewing, and the programs they acquire during development are thought to persist throughout life and transmit to daughter cells (30–33). We are beginning to elucidate some of the tissue-specific metabolic factors that drive regulation of TRM identity. For instance, in the liver, Kupffer cells (the main liver TRM population) rely on the cholesterol synthesis intermediate desmosterol to induce the nuclear receptor liver X receptor alpha (LXRα) (34), which in turn is necessary to induce and maintain Kupffer cell transcriptional identity (34–36). These results are particularly intriguing because previous work suggests that the alternative LXRα ligand oxysterol can, in some instances, induce and potentiate pro-inflammatory gene transcription (37, 38) but in other instances induce cholesterol efflux, repress pro-inflammatory responses, and prevent inflammasome activation (39–41). These findings have important implications for TRM metabolism during homeostasis and resolution, which is discussed further below. Similarly, both splenic red pulp and bone marrow-resident macrophages depend on the porphyrin synthesis product heme, which induces expression of the lineage-defined transcription factor Spi-C *via* degradation of the transcriptional repressor BTB Domain And CNC Homolog 1 (BACH1) (42, 43). However, unlike for Kupffer cells, the source and timing of heme uptake in splenic red pulp or bone marrow-resident macrophages remains unknown. One possibility is that engulfment of dying erythrocytes, which both populations perform millions of times a day (24, 44), provides the key defining source of heme. Alternatively, splenic red pulp macrophages are seeded in a WT1+ stromal network similar to that observed in Kupffer cells (45), which may synthesize and release heme that is subsequently imported *via* the heme transporter HCP1 (SLC46A1) (46, 47).

The above studies also highlight an important consideration: TRMs are generally evenly distributed throughout a complex meshwork of interacting stromal and tissue-specific cells, each of which contribute to the overall nature of the tissue environment (48). Understanding such interactions is understandably complex when considering the exponential nature at which intercellular interactions scale (49). Nonetheless, recent work has illustrated the contribution of resident stroma to the metabolic regulation of TRM identity. For example, a network of cavity-resident stromal cells produce the metabolite retinoic acid *via* transcriptional regulation by Wilms’ Tumor 1 (WT1) (50). Retinoic acid is subsequently required for cavity-resident TRM identity and maintenance through induction of the retinoic-acid-responsive transcription factor GATA6 (16, 17, 50), which in turn regulates the enzyme aspartoacylase (Aspa), necessary for the synthesis of acetyl CoA (51). Conversely, rapamycin-insensitive mTORC2 acts as a negative regulator of GATA6, and its suppression is required for peritoneal TRM differentiation (52). Given that WT1+ stromal cells are a major constituent in two unrelated tissue environments (45, 50), it seems reasonable to conjecture that WT1+ stromal cells are a major non-cell-autonomous regulator of TRM metabolism. In support of this hypothesis, two independent studies observed that the liver also features WT1+ hepatic stellate cells that appear to be important regulators of liver fibrosis and scarring (53, 54). How these cells interact and regulate liver TRM metabolism remains unknown. Collectively, this work serves as a demonstration of the importance of understanding the specific environmental factors that contribute to macrophage identity. Beyond these studies, not much else is known about the metabolic regulation of macrophage identity. Some clues come indirectly from previous studies. For instance, it was previously shown that microglia require cholesterol coupled with TGF-β and IL-34 for identity and phagocytic capacity in mixed cell cultures *in vitro*, which were specifically derived from astrocytes (55). This is consistent with transcriptional analysis of developing microglia indicating significant upregulation of a lipid metabolism program (56). Furthermore, the development of various TRM subsets depend on colony stimulating factor 1 receptor (CSF1R), a receptor for both CSF1 (M-CSF) and IL-34. CSF1R signaling induces macropinocytosis by macrophages (57), suggesting that TRM development and identity depend on lysosomal catabolism of solutes, such as degradation of proteins to generate amino acids (58, 59). Given these findings, it will be interesting to determine how lineage-defining cytokines combine with tissue-specific metabolites to establish and maintain TRM identity.

It is important to note that here, we focus on TRMs that have recently been defined as “resident macrophages” or macrophages that arise in a tissue early on in development. This population is in contrast to monocyte-derived macrophages that can arise in some tissues later in life, during the course of inflammation, or in some cases seed during inflammation and remain after resolution, as reviewed recently (2). This distinction will remain important moving forward not just for basic understanding of macrophage metabolism, but also as a possible therapeutic strategy where these subsets are either beneficial (60) or detrimental (30, 61) to the health of the host. Additionally, most tissues feature multiple types of resident macrophages (2, 5). For example, there are at least three distinct subsets of macrophages in the spleen, defined both by location and physical properties: red pulp, marginal zone, and metallophilic (15). As noted, red pulp macrophages rely on heme metabolism for their identity, whereas neither marginal zone nor metallophillic macrophages depend on heme or Spi-C. Instead, both populations depend on LXRα (62), presumably through metabolic processes similar to Kupffer cells. Given this, it will be important for future studies of TRM metabolism to take into consideration the unique intra-tissue environment differences, including nutrient (63, 64) and oxygen (65, 66) availability, as well as the cell types and debris they engulf (24).

## Macrophage Metabolism During Canonically-Defined Macrophage Polarization

### Pro-Inflammatory Macrophages

**Figure 1:**
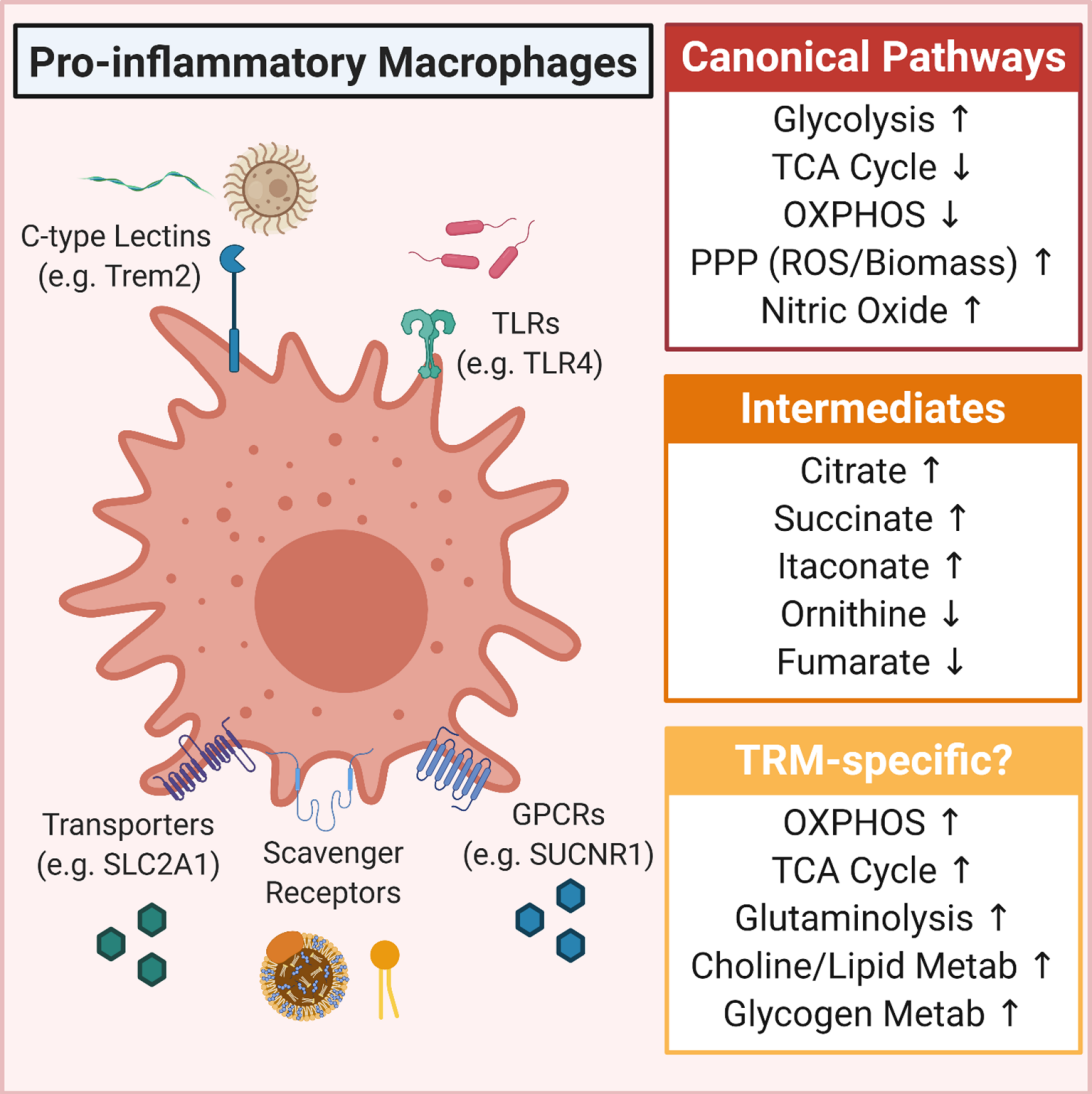
Overview of metabolic pathways induced in pro-inflammatory macrophages. Shown is the canonical pro-inflammatory macrophage metabolic program (Canonical Pathways) as well as some of the more predominantly defined intermediates that accumulate in pro-inflammatory macrophages (Intermediates). Also included are some tissue-resident macrophage (TRM)-specific metabolism observations, including that TRMs in some tissues upregulate OXPHOS and glutaminolysis, that lipids (such as oxidized phosphoplipids and high/low-density lipoproteins) can drive a hyper-metabolic state, and in some contexts, glucose is shunted into glycogenesis.

Danger-associated molecular patterns (DAMPs) and pathogen-associated molecular patterns (PAMPs) are at the core of sterile inflammation and microbial infection, respectively (67, 68). Both DAMPs and PAMPs are known to induce an inflammatory response coordinated by innate immune cells, including monocytes, macrophages and dendritic cells, and that uncontrolled chronic inflammation is associated with a myriad of human diseases (68). Notably, the growing field of immunometabolism has underscored how cellular metabolic reprogramming is at the center of this coordinated inflammatory response (69–71). Work more than three decades ago hinted at metabolic changes in macrophages exposed to microbial ligands (72–74), but through a series of elegant *in vitro* experiments, we have come to better understand the precise nature of these metabolic changes. The specifics of these metabolic changes are detailed in several fantastic reviews. Here, we briefly outline the broader metabolic switch that occurs in canonically pro-inflammatory macrophages. Typically, a naïve macrophage, defined as a macrophage differentiated from bone marrow stem and progenitor cells cultured in high glucose (~20-25mM), normoxic oxygen (~21% or ~160 mmHg O_2_), and the differentiation cytokine(s) M-CSF/GM-CSF, relies primarily on the tricarboxylic acid (TCA) cycle and mitochondrial respiration to generate much of the ATP used for basic cellular processes. However, seminal work from O’Neill and colleagues demonstrated that activation with the PAMP lipopolysaccharide (LPS) induces macrophages to undergo a metabolic switch from a state of high oxidative phosphorylation (OXPHOS) to a highly glycolytic state (75). Accumulation of glycolytic intermediates and byproducts further support primary functions of activated macrophages, including providing glucose-6-phosphate to the pentose phosphate pathway (PPP) which contributes to both building of biomass and regulating the redox state, as well as induce the production of immune effectors such as itaconate and IL-1β [summarized in depth here (69)]. Building from this work, numerous studies have demonstrated the importance of this metabolic switch for key inflammatory macrophage functions, including bacterial phagocytosis, production of pro-inflammatory cytokines, synthesis of antimicrobial peptides, and generation of reactive oxygen species (ROS) (76–79). Collectively, this work demonstrates that quiescent macrophages undergo striking metabolic changes in support of one of its core immunological roles as a defender against invading pathogens (**Figure 1**).

### Pro-Resolving Macrophages

Together with defense against pathogens, macrophages are key orchestrators of the post-injury response (24, 29, 80). This response, canonically known as the wound healing or resolution response, is in many ways the mirror image of the canonical pro-inflammatory response, including broad metabolic changes (29, 81). Generally speaking, these ‘pro-resolving’ macrophages are derived using the same base culturing conditions as pro-inflammatory macrophages, but naïve macrophages are stimulated with IL-4 instead of LPS (or IFNγ). In contrast to canonical pro-inflammatory macrophages, pro-resolving macrophages remain significantly increase TCA cycle activity and OXPHOS, with less dependence on glycolysis, as well as exhibiting significantly increased fatty acid oxidation (FAO) (82, 83). Though it is important to note that the ‘importance’ of glycolysis remains in debate, and likely depends on the nature of the culture conditions (e.g. glucose and oxygen availability) (83, 84). The observed increase in TCA cycle activity is the result of IL-4-induced PPARγ transcriptional activity, which in turn modulates expression of key enzymes, respiratory chain function, and glutamine metabolism (85–87). An additional benefit of enhanced TCA cycle activity is the accumulation of α-ketoglutarate (α-KG), as α-KG was shown to blunt canonical pro-inflammatory cytokine production *via* suppression of inhibitor of nuclear factor kappa-B kinase subunit beta (IKKβ) and to induce canonical anti-inflammatory cytokine production *via* activation of the histone demethylase Jumonji domain-containing protein D3 (JMJD3) (88).

**Figure 2:**
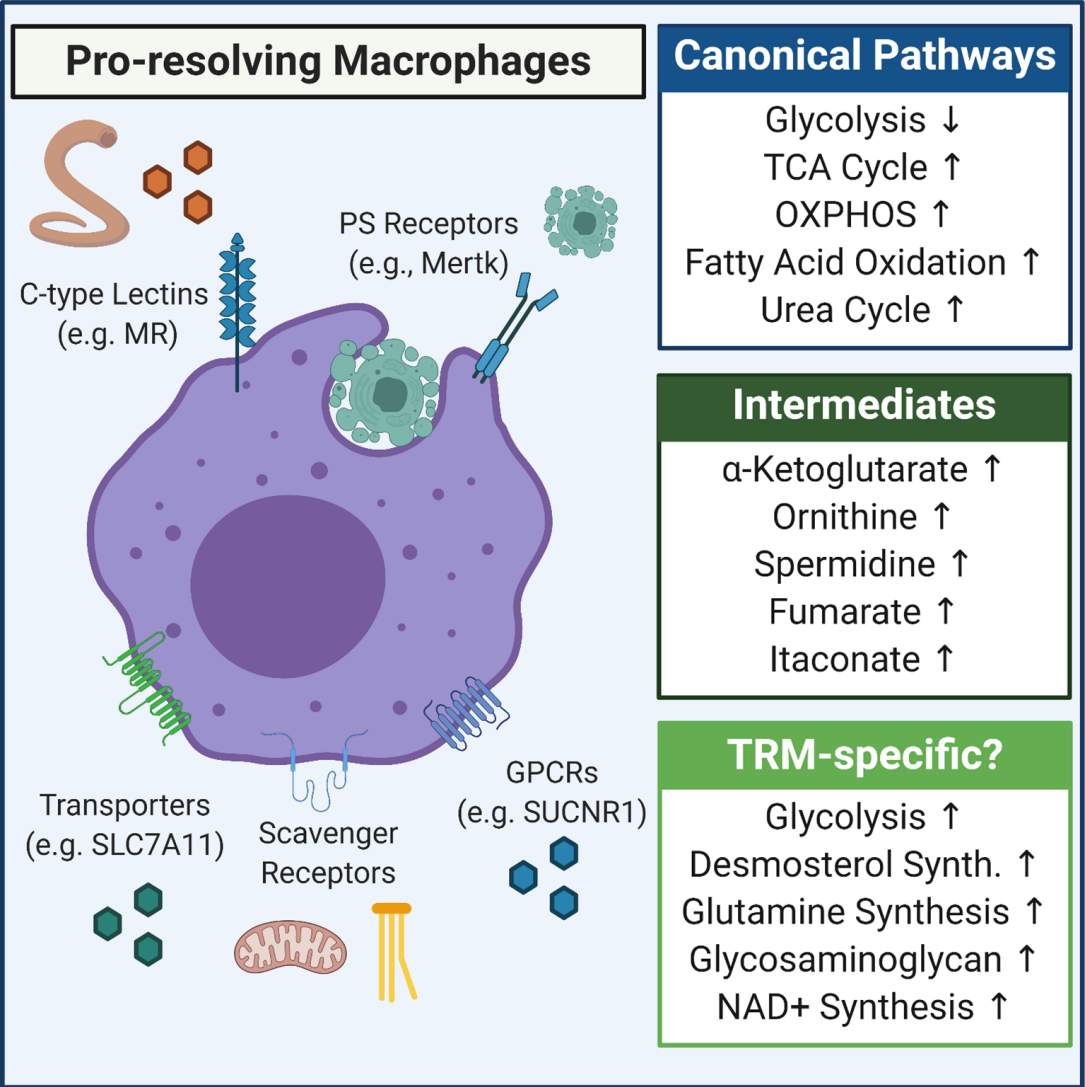
Overview of metabolic pathways induced in pro-resolving macrophages. Shown is the canonical anti-inflammatory/pro-resolving macrophage metabolic program (Canonical Pathways) as well as some of the more predominantly defined intermediates that accumulate in pro-resolving macrophages (Intermediates). Also included are some tissue-resident macrophage (TRM)-specific metabolism observations, including that apoptotic cells and/or cytokine (e.g., IL-4/IL-13) induce upregulation of glycolysis, that succinate accumulates and signals *via* SUCNR1 to drive inflammation resolution, and that novel pathways such as desmosterol synthesis are induced in specific tissues, such as the CNS.

One final aspect of this metabolic program worth highlighting, given its macrophage response-defining role, is the importance of arginine and polyamine metabolism (29). In mouse macrophages, it is generally thought that arginine is catabolized *via* two distinct routes: *via* an inducible nitric oxide synthase (iNOS) catalyzed dehydration reaction that produces citrulline and nitric oxide or by an arginase 1 (Arg1) catalyzed hydrolysis reaction that produces ornithine and urea (77). The former reaction is thought to be a key component of the pro-inflammatory macrophage response to pathogen invasion (78), whereas the latter is thought to be a key step to the generation of pro-resolving macrophages (81). Ornithine, and its conversion to putrescine *via* ornithine decarboxylase (ODC), as part of polyamine synthesis, is essential for multiple facets of the induction and maintenance of pro-resolving macrophages (89) (**Figure 2**).

### Considerations Moving Forward

Several excellent reviews provide a survey of our general understanding of the metabolic changes that arise in macrophages in response to IL-4 stimulation (29, 69, 71, 81). Although IL-4 has served as an important model for pro-resolving macrophage metabolism, how these macrophages arise *in vivo* remains an intriguing area of investigation. For instance, one likely situation occurs after the clearance of a pathogen or during the resolution of a sterile injury. Those macrophages that do not die as part of the immune response (e.g., *via* pyroptosis) switch to a pro-resolving state. A less obvious example arises in the case of type 2 immunity, where the specific infection (e.g., helminths) directly induces a pro-resolving phenotype in macrophages. Intriguingly, macrophages are also thought to assume a wound healing phenotype during homeostatic apoptotic cell clearance (termed ‘efferocytosis’). Recent work suggests that, at least in some contexts, both IL-4 (or IL-13) and exposure to apoptotic cells is required to induce a pre-resolving macrophage response *in vivo (* 90 *)* (**Figure 2**). Thus, it will be important to understand the shared and unique metabolic features of TRMs in each of these contexts.

Several controversies related to macrophage metabolic (re)programming remain unresolved. This is likely due, in part, to supraphysiological culturing conditions. The use of *in vitro* systems will remain the key to understanding macrophage metabolism at a mechanistic level, therefore solutions as outlined previously are warranted. An additional complication relates to how metabolic pathways are perturbed. The use of small molecule inhibitors, RNA interference, and increasingly genetic deletion, to perform ‘down’ assays has informed much of our understanding. However, it has also likely led to conflicting results, for instance due to off-target effects in the case of small molecules or genetic compensation in the case of genetic deletion. Furthermore, it remains controversial how different types of macrophages utilize these canonical metabolic programs and how similar (or not) murine and human macrophage metabolism are. For example, whether differential arginine catabolism *via* iNOS and ARG1 is relevant for inflammatory and resolving functions in human macrophages remains highly debated (91, 92).

The use of complimentary approaches, including the incorporation of more ‘up’ assays *in vitro* and *in vivo*, will allow the field to understand these important questions in greater detail.

## Tissue-Resident Macrophage Metabolism During Inflammation

As noted, much of our understanding regarding metabolic control of macrophage immunity has come from *in vitro* studies. Recently, groups have begun to extend these findings into *in vivo* settings, particularly into physiologically relevant models of human disease (93). In this section, we will first summarize the state of *in vivo* macrophage immunometabolism studies in the context of inflammation. We will particularly focus on the most recent findings of how metabolic reprogramming impacts and shapes the functions of inflammatory macrophages *in vivo* (**Figure 1**). However, it is important to note that, aside from work on microglia, the majority of *in vivo* macrophage metabolism research does not necessarily delineate TRM subsets, which will be important given there is likely spatiotemporal regulation of macrophage metabolism, as seen in the tumor microenvironment (94).

As briefly discussed above, aerobic glycolysis is a key feature of inflammatory macrophages. Interestingly, byproducts of glycolysis are repurposed to support additional metabolic pathways that drive important macrophage functions (69). Most notably, LPS-activated macrophages accumulate TCA cycle intermediates that possess immunoregulatory properties (9). The metabolite succinate was identified as an inflammatory metabolite that induces IL-1β transcription *via* HIF-1α (75). In these early studies, both macrophage-specific absence of *Hif1a*, or treatment with the GABA-shunt inhibitor vigabatrin (which prevents succinate production) protected from endotoxin-induced sepsis (75). Interestingly, a recent study from Cardaci and colleagues found that supplementation with D-mannose given intraperitoneally (i.p.) or *via* water/oral gavage protected mice from endotoxin-induced sepsis and dextran sodium salt (DSS)-induced colitis, respectively (95). Mechanistically, D-mannose treatment acts to competitively inhibit glycolytic production of succinate, triggering a counter-intuitive increase in mannose-6-phosphate levels, possibly owing to low level expression of the enzyme phosphomannose isomerase (MPI) in macrophages (95). Additionally, inflammatory macrophages release succinate into the extracellular environment, which then triggers an autocrine/paracrine amplification loop *via* signaling through the succinate receptor SUCNR1/GPR91, increasing IL-1β production and exacerbating joint swelling in a mouse model of arthritis (96). Intriguingly, synovial joint macrophages were recently described as a locally-renewing TRM population that provide a key barrier in the synovial joint that, unlike infiltrating monocyte-derived macrophages, act to prevent/dampen inflammation (97). How local succinate production acts on each of these populations of synovial joint macrophages is an intriguing question, especially given succinate can have opposing actions depending on the cell type and context. For instance, inflammatory microglia-derived succinate was shown to instruct neural stem cells, *via* GPR91, to secrete prostaglandin E2 (PGE2). PGE2 in turn scavenged excess succinate, resulting in decreased macrophage activation and alleviated inflammation in experimental autoimmune encephalomyelitis (EAE), a mouse model of CNS inflammation (12). This work highlights how dynamics in the specific tissue microenvironment can lead to unexpected results *in vivo*.

As highlighted in recent work from the O’Neill and Artyomov labs, the TCA cycle intermediate itaconate has emerged as a model ‘immuno-metabolite’, given its essential roles for both pro-inflammatory and pro-resolving macrophage function (98). On one hand, itaconate (as well as the cell-permeant derivates dimethyl itaconate or 4-octyl itaconate) acts as a potent anti-inflammatory modulator by suppressing production of various inflammatory cytokines IL-1β, IL-6, and the type I interferon IFNβ. Mechanistically, itaconate (or its derivatives) functions through multiple mechanisms, including inhibiting glycolytic enzyme activity, activation of the oxidative stress response transcription factor NRF2, modification and inhibition of the NLRP3 inflammasome, and induction of ATF3-mediated regulation of IκBζ (99–105). How endogenous itaconate directly affects TRMs *in vivo* is less clear, especially because itaconate appears to directly inhibit pro-resolving macrophage differentiation (81, 106), and enhance IFNβ production in response to LPS (104). Additionally, *in vivo* studies have either used mice globally-deficient in the enzyme necessary to produce itaconate from citrate (immune responsive gene 1, Irg1) or delivered exogenous itaconate (or one of the cell-permeant derivates), making conclusions about TRMs versus other tissue-resident cells capable of utilizing exogenous itaconate challenging (99). Nevertheless, absence of Irg1 or delivery of exogenous itaconate derivatives were shown to dampen LPS-induced sepsis (100), alleviated skin pathology in a mouse model of psoriasis (99), limits myocardial infarct size during ischemia-reperfusion injury (103), and suppressed inflammation in urate crystal-induced peritonitis (102).

This seminal series of studies triggered subsequent exploration of immunoregulatory roles of primary metabolites. For instance, Fitzgerald and colleagues found that the TCA cycle intermediate fumarate inhibits pyroptosis *via* succination of the cysteines in the pyroptosis effector protein gasdermin D (GSDMD). Indeed, treatment of mice with Tecfidera, the fumarate derivative dimethyl fumarate (DMF), alleviated symptoms in both EAE and Familial Mediterranean Fever mouse models as well as providing a mechanistic explanation for the immunomoulatory activity of Tecfidera in MS patients (107). Unlike in classical LPS-activated macrophages, ‘hyperactivation’ of macrophages is induced by endogenous DAMPs, such as oxidized phospholipids (108). Hyperactivated macrophages also exhibit hyperactive metabolic activity, relying on glycolysis, oxidative phosphorylation, and glutamine catabolism (109). Hyperactive macrophages accumulate the TCA cycle intermediate oxaloacetate, likely as a result of multiple substrates feeding the TCA cycle, which exacerbates IL-1β production (109). This phenomenon was observed in plaque-associated macrophages in *Ldlr*- and *Apoe*-deficient mice fed high fat diet, which could be prevented by inhibiting either glutaminase or ATP citrate lyase (109, 110). The atherosclerotic plaque is likely a unique microenvironment itself, as the metabolic state of plaque-associated macrophages (also known as Mox macrophages) seems to be characterized by several unique facets (111), reviewed in greater detail elsewhere (26). We highlight Mox macrophages, however, because recent work suggests that adipose TRMs in lean adipose tissue exhibit metabolic characteristics similar to Mox macrophages, possibly because of differences in individual oxidized phospholipid species (112). Contrarily, adipose TRMs isolated from obese mice show characteristics of canonical pro-inflammatory macrophages such as the IL-1 pathway (113).

Unsurprisingly, the TCA cycle is not the only metabolic pathway relevant in inflammatory macrophages. For example, LPS induces translocation of histone deacetylase 3 (HDAC3) to the mitochondria which subsequently deacetylates and inactivates the alpha subunit of hydroxyacyl-CoA dehydrogenase (HADHA), an enzyme essential for fatty acid oxidation (FAO), which was necessary for IL-1β production (114). Myeloid cell-specific deletion of HDAC3 lessened the severity of both LPS-induced sepsis and diet-induced type 2 diabetes, the latter of which appeared to be a direct effect on adipose TRM metabolism (114). Additionally, LPS stimulation upregulates the choline transporter CTL1 (SLC44A1), facilitating increased uptake and generation of phospho-choline species (*via* the Kennedy Pathway), altering mitochondrial phospholipid and sphingolipid species profiles, and contributing to NLRP3 inflammasome activation (115). Inhibition of choline kinase lessened the inflammation in both LPS-induced sepsis and a urate crystal-induced gout model, as well as reduced splenomegaly and size in a mouse model of Muckle-Wells syndrome (115). The changes in macrophage lipid metabolism observed in response to LPS stimulation are consistent with two recent studies of lipid metabolism in inflammatory macrophages (116, 117). Finally, one carbon, glycogen, and PPP metabolism were shown to regulate numerous aspects of the *in vivo* inflammatory macrophage response to LPS (118–120). Taken together, it is clear that TLR signaling induces a plethora of metabolic changes in macrophages *in vivo*. It will be interesting to see how different TRM populations respond to the combination of inflammatory stimuli and unique tissue environment factors, such as the unique factors in adipose tissue highlighted above. This is exemplified by a recent study which found that alveolar macrophages, who reside in a glucose-restricted environment, rely on OXPHOS and not glycolysis to mount an inflammatory response to acute inflammation induced by LPS or to influenza infection (121). Understanding how alveolar macrophages are able to produce the requisite inflammatory cytokines in the absence of glycolytic reserve may also elucidate novel immunoregulatory metabolites. Indeed, elucidating these unique metabolic regulatory pathways, both in alveolar macrophages but also stromal and immune cells that regularly interact with alveolar macrophages, could prove important for managing the devastating consequences of chronic lung disease [the lung metabolic environment is reviewed in detail elsewhere (122)]. As has likely become apparent, the vast majority of *in vivo* studies of inflammatory macrophage metabolism use variations of the LPS-induced sepsis model. Naturally, the immunometabolism of TRMs in response to tissue-relevant infections is an important and rapidly growing area of research. For instance, alveolar macrophages rely on different metabolic programs depending on the type of infection (121, 123). Additionally, emerging evidence suggests that commensal microbes contribute to metabolic reprogramming of intestinal TRM populations (124–126). Given that tissue-specific infectious defense is a core function of TRMs, moving beyond LPS will certainly provide new insights into unique TRM metabolic programs used during infectious immunity.

Of the long-lived TRM populations, microglia metabolism is arguably the most studied. Beginning with seminal work from the Barres lab, microglia researchers realized very early on that the CNS environment, including the debris that microglia engulf, the abundant (and unique) lipid species present, and the types of damage that occur, are equally important to informing microglia metabolism as intrinsic programming (63, 64, 127, 128). In some ways, microglia behave similarly to other macrophages, such as the metabolic shift away from TCA Cycle activity/mitochondrial ATP generation towards increased glucose uptake (via upregulated GLUT1/SLC2A1) and breakdown into lactate as well as increased mTOR activity, in response to TLR ligands or inflammatory cytokine stimulation (63, 128, 129). These microglia, termed reactive microglia, are particularly clinically relevant because they have been shown to arise in chronic neurodegenerative diseases, such as Alzheimer’s Disease (AD) (130–135). Interestingly, microglia that populate plaques in AD, termed disease-associated microglia (DAMs) (136), appear to transition from an initial glycolytic, reactive state into an apparent hypometabolic state with significantly altered mTOR activity, which is possibly related to alterations in the phagocytic and/or lipid-sensing function of the AD risk-associated triggering receptor on myeloid cells 2 (TREM2) (130, 131, 134). However, microglia also exhibit potentially unique properties, including the ability to utilize alternative energy sources, which may prove especially important given the restrictive CNS parenchyma (63, 137). An additional unique aspect of the CNS that is likely driving microglia metabolism is the presence of diverse lipid species, the ramifications of which have recently been discussed extensively elsewhere (128). Ultimately, it seems apt to look to the various studies of microglia metabolism as a model for how we should study peripheral TRM metabolism moving forward.

## Metabolism of Homeostatic and Pro-Resolving Tissue-Resident Macrophages

As previously discussed, numerous studies suggest that pro-resolving macrophages, typically defined as IL-4-stimulated, rely extensively on TCA cycle activity and OXPHOS for ATP generation, anti-inflammatory cytokine production, and the establishment of the canonical wound healing response (**Figure 2**). Intriguingly, the relative importance of glycolysis to IL-4-stimulated macrophage polarization remains controversial (82–84, 138). It is possible that there is some level of net increase in glycolytic flux induced by transitioning from a naïve macrophage to a stimulated state (138). It is also possible that the variation in macrophage response depends on the levels of insulin/insulin-like growth factors derived from serum as well as the dynamics of glucose availability, which can affect glucose transporter expression and thus the ability and reliance on glucose uptake (139–142). Interestingly, macrophages engulfing apoptotic cells (termed ‘efferocytosis’), which is a canonical pre-resolving macrophage function, exhibit dynamic changes in metabolic state depending on the stage of clearance. Specifically, receptor engagement of apoptotic cells induces glucose uptake and aerobic glycolysis which is necessary for rapid ATP generation to polymerize actin and promote internalization of apoptotic cells (143). On the other hand, internalization (and presumably digestion) of apoptotic cells induces fatty acid oxidation (FAO) and OXPHOS, which is required for specific aspects of the pro-resolving response including IL-10 (144). This ultimate dependence on FAO for pro-resolving macrophage function was also observed in peritoneal TRMs in two models, IL-4/IL-4R complex injection and *Heligmosomoides polygyrus (H. polygyrus*) *(* 86). However, in the latter case, the primary source of lipids was exogenous triacylglycerol instead of apoptotic cells. What the source of lipids are, whether different TRMs require FAO to fuel canonical macrophage function, and if the oxygen and substrate availability unique to different tissues changes pro-resolving TRM function (145), remains unknown.

Pro-resolving macrophages are canonically characterized by the preferential hydrolysis of arginine into ornithine and urea *via* the enzyme Arg1. Ornithine, together with its essential role in detoxification of ammonia in the urea cycle, is also the entry point into the polyamine synthesis pathway. Interestingly, Puleston and colleagues found that pro-resolving macrophages depend on the polyamine biosynthesis pathway to support TCA Cycle and OXPHOS (89). Intriguingly, the polyamine synthesis product spermidine was consumed in a novel post-translational modification termed ‘hypusination’ – or the addition of amino acid hypusine. The only known eukaryotic protein to be modified by hypusine is translation initiation factor 5A (eIF5A), which in macrophages was necessary for the expression of a subset of mitochondrial respiration proteins (89). Importantly, the IL-4/IL-4R and *H. polygyrus* models of pro-resolving peritoneal TRM function revealed the importance of eIF5A hypusination *in vivo*. Similar to *in vivo* findings related to lipid sources for FAO, TRMs may derive arginine, ornithine, or spermidine from various sources, including *via* regulated release by (23) or engulfment of apoptotic cells (146).

There is an emerging body of work exploring immune and non-immune homeostatic functions of TRMs, including local and systemic control of organismal metabolism, metabolite release to promote tissue regeneration, and maintenance of tissue homeostasis *via* organelle clearance. For example, previous studies suggested that liver inflammation, mediated by generation of inflammatory liver TRMs, underlies the presentation of obesity-induced metabolic disease including altered hepatic glucose production, progression to steatohepatitis, and liver fibrosis (147). However, a recent report presents striking contradictory evidence from flies, mice, and human patients (148). Specifically, they found that obesity-induced insulin resistance does not induce pro-inflammatory activation of liver TRMs, but rather produce insulin-like growth factor-binding protein 7 (IGFBP7) which acts as a non-inflammatory immune cell-derived metabolite to induce lipogenesis and gluconeogenesis *via* insulin receptor signaling. Interestingly, variations in the IGFBP7 isoform appeared to differentially bind the insulin receptor, possibly explaining why some obese patients develop insulin resistance whereas others do not (148). Importantly, this work highlights the possibility of a TRM-specific tissue metabolic circuit functioning independent of canonical immune pathways (149). It is important to highlight that, although the finding that liver TRMs (specifically Kupffer cells) remain ‘non-inflammatory’ during obesity was recently confirmed, Scott and colleagues found that Kupffer cells are lost over time in the obese mouse, replaced by bone marrow-derived macrophages (150). Intriguingly, a fraction of these macrophages express osteopontin and reside in regions of increased desmin, suggesting that these infiltrating macrophages are contributors to liver fibrosis. One final note, unlike the previous study, Scott and colleagues observed that *Igfbp7* expression was restricted to the non-hematopoietic compartment, which is consistent with the observed loss of Kupffer cells (150). Whether these differences are simply differences in the model used, or an interesting observation of the changes in tissue dynamics over the course of the disease, remains an important point of future investigation. Nevertheless, it will be interesting to explore how systemic metabolic stress, an ever-growing reality in many countries (151), affects various TRM populations, in particular how TRMs fight to maintain organismal homeostasis within biologically safe parameters and why/how those safeguards fail (152, 153).

One possibility is that TRMs use different transporters and G-protein coupled receptors (GPCRs) to respond to metabolites present in the extracellular milieu. As an illustration, it was recently shown that adipose TRMs respond to extracellular succinate *via* the GPCR succinate receptor 1 (SUNCR1) (153). Unlike its role in certain contexts, succinate signaling through SUNCR1 maintained metabolic homeostasis across multiple contexts, including in mice fed normal chow, fed high-fat diet, and exposed to cold temperature (a model of adipose tissue-browning). Importantly, these findings also correlated to comparisons between lean and obese patients (153). This is a striking example of how the function of an immunoregulatory metabolite can vary depending on the tissue and context. As discussed below, emerging methods and technologies will facilitate a better understanding of the *in situ* dynamics of TRM metabolism, including intercellular interactions. In a recent study, Crawford and colleagues used stable isotope tracing-based untargeted metabolomics to investigate how hepatocyte-derived substrates (glucose and ketone bodies) are metabolized by liver TRMs (154). This method allowed for an extensive characterization of liver TRM metabolic pathways used under various contexts, including elucidating a novel role for acetoacetate oxidation in liver TRMs required to fuel the glycosaminoglycan pathway and prevent of liver fibrosis (154).

Two recent papers highlight the importance of the specific tissue microenvironment on the canonical TRM function of tissue repair and regeneration. In the first study, Berardi, Mazzone, and colleagues elucidate a system of muscle regeneration by which muscle TRMs act as sensors of glutamine availability which is a key determinant of successful regeneration. Specifically, upon muscle injury or in aged muscle, glutamine levels fall in the extracellular milieu, inducing TRMs to switch from glutamate oxidation (via glutamate dehydrogenase 1, GLUD1) to increased glutamine synthesis (via glutamine synthetase, GS) and release (155). Satellite cells, the multipotent muscle stem cell, consume TRM-derived glutamine *via* the transporter SLC1A5 which facilitates their proliferation and differentiation into mature muscle. This circuit was tunable, allowing for both improved and impaired muscle regeneration across multiple *in vivo* models (155). In the second study, Nave, Saher, and colleagues report that microglia-mediated CNS repair depends on the synthesis of desmosterol (instead of the sterol synthesis end-product cholesterol) from the engulfed myelin (156). Desmosterol, in turn, signals *via* the liver X receptor (LXR) to induce lipid efflux and secretion of reparative factors to support remyelination. Indeed, inducing sterol synthesis promoted quicker remyelination *in vivo (* 156 *)*. Conceptually, each tissue features unique accessory and primary cell types, as highlighted in the above studies, that function collectively to perform the required functions of that tissue. The types of material transferred to TRMs is not restricted to metabolites, as two recent studies found that mitochondria are transferred to or cleared by TRMs in the adipose tissue and the heart as part of tissue homeostatic processes (157, 158). Collectively, these findings highlight exciting and emerging areas of TRM metabolism and biology.

As noted in the beginning of this review, a TRM’s life-long environment, or *habitare*, is the sum total of factors that are experienced. Two recent reviews make the conjecture that a TRM’s *habitare* includes time spent in residence. Although this is a relatively understudied aspect of TRM biology, there is at least some evidence to suggest that TRM metabolism is affected during aging. In two separate studies, the Andreasson lab details how aging peritoneal TRMs and microglia exhibit altered, although potentially reversible, changes in metabolism that accompany perturbed homeostatic function (159, 160). In the first study, the authors show that macrophages synthesize nicotinamide adenine dinucleotide (NAD+) *via* the kynurenine pathway (KP), which is necessary for mitochondrial respiration and canonical pro-resolving macrophage function (159). Intriguingly, the ability to synthesize NAD+ *via* the KP is decreased in aging peritoneal TRMs but was reversed *via* overexpression of the KP pathway enzyme nicotinate-nucleotide pyrophosphorylase (QPRT) or *via* exogenous delivery of the NAD+ precursor nicotinamide mononucleotide (NMN) (159). In the second study, peritoneal TRMs and microglia exhibited an age-dependent perturbation in OXPHOS resulting from increased PGE2-mediated glycogenesis. Increased PGE2 signaling *via* its cognate receptor EP2 resulted in a maladaptive pro-inflammatory state that altered mouse cognition, which was reversible when inhibited *in vivo (* 160 *)*.

One final point worth highlighting when discussing the time TRMs spend in residence is the effect that chronic, low-grade perturbations have on TRM identity. This is best exemplified by recent studies of Kupffer cells in mouse models of metabolic-associated fatty liver disease (MAFLD). Intriguingly, Kupffer cell numbers and/or identity is lost during the course of the MAFLD sequelae non-alcoholic steatohepatitis (NASH) (62, 150, 161, 162). These cells are, at least in part, replaced by monocyte-derived macrophages that appear to seed the liver long-term and assume transcriptional programs similar to those of their long-term TRM counterparts. This is intriguing because these newcomers, despite exhibiting transcriptional, epigenetic, and functional similarities to their long-lived Kupffer cell counterparts (34, 150, 161), appear to have different responses to lipids (including triglycerides) and contribute to NASH progression differently (161, 162). For instance, on one hand, monocyte-derived liver TRMs appear to less efficiently promote storage of triglycerides and exacerbate liver fibrosis (161). On the other hand, mice lacking CCR2, which prevented monocyte-derived liver TRM seeding, resulted in *increased* liver fibrosis (162). These seemingly contradictory results may actually represent functional evolution of monocyte-derived liver TRMs, ultimately supporting the notion that the time spent in a particular tissue, particularly as the nature of that tissue changes, informs the function and metabolic state of the TRM. Taken together, it is apparent that future work will need to take into consideration the multiple factors that constitute a TRM’s *habitare*, especially time spent in residence and the changing nature of that residence.

## Novel Techniques to Study Tissue-Resident Macrophage Metabolism

The majority of what we know about macrophage metabolism comes from a combination of *in vitro* mechanistic and *in vivo* genetic/small molecule targeting studies. These approaches will continue to be essential moving forward, and the utilization of methods for modeling the appropriate tissue environment and differentiating unique TRM subsets will further advance our understanding of TRM metabolism (163, 164). However, the emergence of better methods and technologies will allow for a holistic understanding of TRM metabolism, including at the single cell level *in situ*. For an in-depth overview of single cell analysis of cellular metabolism, including statistical methods for analyzing single cell RNA sequencing, we recommend the perspective from Artyomov and Van den Bossche (165). Here, we outline some of the methods and technologies used to study metabolism *in vivo* that have been or could easily be applied to study TRM metabolism.

*Raman Spectroscopy and Fluorescence Imaging*. Molecules, especially complex metabolites, exhibit numerous unique properties that can be exploited to study cell metabolic processes. These approaches generally fall under the umbrella of molecular imaging. One such property is the Raman effect (or Raman scattering), which is the inelastic scattering of photons when a molecule is excited by a laser beam. Raman spectroscopy (RS) imaging combines information obtained from this property together with light microscopic information to perform label-free, non-destructive analysis of cellular metabolism. Proof-of-principle studies have shown that RS imaging can be used to image macrophage heme metabolism (166) as well as macrophage fatty acid and lipid metabolism (167). Additionally, deuterium can be used as a stable isotope label of a metabolite of interest, allowing for both analysis of intracellular distribution and metabolic flux in macrophages (167). RS imaging exhibits high sensitivity and specificity for metabolite detection and is capable of rapid imaging speeds (167). On the other hand, despite improved technology and methods, RS imaging continues to suffer from relatively low spatial resolution (167). Despite this caveat, RS imaging still has the potential to analyze cellular metabolism *in situ* when combined with confocal, multiphoton, or lightsheet microscopy (168–171).

Cellular metabolism is modulated, in large part, by the redox state of the cell. One major determinant of this redox state is the ratio of oxidized and reduced forms of nicotinamide adenine dinucleotide (NAD+/NADH) and nicotinamide adenine dinucleotide phosphate (NADP+/NADPH). Classically, NAD+ is a key cofactor for ATP production *via* glycolysis (in the cytosol) and OXPHOS (in the mitochondria), whereas NADP+ (and importantly its reduced form NADPH) is a key cofactor for the synthesis of the antioxidant glutathione (GSH) and for anabolic metabolism pathways, such as lipid synthesis. Naturally, there has been much effort put into developing methods for real-time monitoring of NAD(P)/NAD(P)H pool sizes (172, 173), and more recently subcellular localization (174), using genetically-encoded fluorescent protein sensors in live cells and transparent model organisms. Recently, two independent groups reported the development of ratiometric fluorescence probes that allow for specific monitoring of NADP/NADPH (175, 176). The first, Apollo-NADP+, relies on the principle of fluorescence anisotropy (also known as fluorescence polarization), that occurs because of the Förster resonance energy transfer (FRET) that occurs between homologous fluorescence proteins. Fluorescence anisotropy, similar to the anisotropy exhibited by tissues and exploited by magnetic resonance, is when the emitted light from an excited fluorescent protein exhibits unequal polarization across spatial dimensions and has become an important tool for studying protein-protein interactions (177, 178). Apollo-NADP+ functions by monitoring the single photon-induced anisotropy of monomeric (inactive) glucose-6-phosphate dehydrogenase (G6PD) and dimeric (active) G6PD, based on the knowledge that NADP+ binding to G6PD stabilizes G6PD dimers (179). Importantly, Apollo-NADP+ is a direct metric of NADP/NADPH levels and a more rapid indicator of cellular redox state than fluorescent protein reporters of hydrogen peroxide (176). The second collection, termed iNap (175), is a series of pH-resistant, ratiometric sensors based on the circularly permuted YFP NADH/NAD sensor, SoNar (180). Both iNap and SoNar function by fusing the NAD(H) binding domain Rex from Thermus aquaticus, with iNap including modifications to Rex to facilitate binding to NADP(H) instead of NAD(H). Excitingly, both SoNar and iNap were shown to be capable of monitoring subcellular pools in live cells *in vitro* (175, 181) as well as single cell metabolic function *in vivo (* 175, 182). Finally, as a proof-of-principle, the authors used iNap to demonstrate the ability to rapidly assess NADPH pool size decrease in response to LPS+IFNγ stimulation of macrophages, consistent with the knowledge that inflammatory macrophages consume NADPH to produce superoxide free radicals *via* NADPH oxidase activity.

The fluorescent protein sensors described above are just a subset of the numerous reporters developed to monitor cellular metabolism in live cells [reviewed in (172)]. As noted, *in vivo* use of fluorescent protein reporters of metabolic activity has primarily focused on model organisms that can be imaged easily. However, proof-of-principle studies in mice do exist, for instance with both the SoNar and iNap reporters described above in muscle cells of young and aged mice. One limitation relates to how to introduce a genetically-encoded reporter. Both muscle and neurons (another regularly investigated cell type), for instance, are easily electroporated or transduced with adeno-associated viruses (AAV). Macrophages, especially TRMs that are not replenished by bone marrow-derived progenitors, are generally resistant to such approaches. The natural solution is to introduce these reporters into mice using a conditionally-induced transgene strategy. This has historically been a cost-ineffective strategy: however optimized protocols for using CRISPR/Cas9-induced homology-directed repair to introduce such transgenes into fertilized oocytes has made such an endeavor more realistic. It would be interesting to combine NAD/NADH or NADP/NADPH reporter mice together with recently described tools to manipulate these ratios *in vivo* (183–186) to study TRM redox state and metabolism *in vivo*.

It is also worth highlighting that NADH (and NADPH) exhibits fluorescence (460 ± 50 nm) when excited by an ultraviolet laser (340 ± 30 nm) or a tunable infrared two-photon laser (750 ± 30 nm) (137, 187, 188). When excited, free NADH has a much shorter fluorescence lifetime than bound NADH, which can be quantified using fluorescence lifetime imaging microscopy (FLIM). Indeed, as noted above, MacVicar and colleagues used two-photon FLIM to demonstrate that microglia can adapt to hypoglycemia by using glutamine to maintain NAD(P)H levels (137). In a separate study, Keely and colleagues provide proof-of-principle evidence that FLIM can be used to monitor NAD(P)H and FAD levels in stromal-resident macrophages, in this case tumor-associated macrophages in the mammary tumor microenvironment (188). Both of these studies were unable to distinguish between NADH and NADPH because of their similarities. A previous study highlighted FLIM methods that distinguish between NADH and NADPH (187). Given the distinct functional importance, future work should incorporate this method. It will be interesting to see if different TRM populations exhibit unique NAD(P)H profiles *in situ*.

The methods detailed in this review require special equipment or expertise, therefore methods that are more readily available are warranted. Flow cytometry is a gold standard immunological technique, with instruments that are more readily available and have gone down significantly in price. Two recent studies detail methods that use high-throughput multi-parameter flow cytometry to either 1) specifically detecting important metabolic proteins including rate-limiting enzymes and transporters (189) or 2) quantify puromycin uptake as a surrogate of cellular metabolism (190). Unfortunately, both of these methods could possibly require time-consuming and cumbersome tissue digestions to release TRMs, which could ultimately affect the metabolic state of the cell (191). Similar to RNA sequencing-based approaches, these methods are restricted in their ability to directly assess metabolic activity. Nevertheless, these methods provide additional tools to the immunologist’s repertoire that are easy to adapt to assess TRM metabolism.

One final fluorescence-based method that is important to discuss involves the combination of histocytometry and enzyme histochemistry to probe the enzymatic activity of common metabolic pathways *in situ (* 192). Specifically, Haschemi and colleagues took advantage of the knowledge that intact tissues fresh-frozen in a preservative such as OCT remain metabolically active and performed nitroblue tetrazolium chloride (NBT)-based enzymatic essays on tissue sections to assess the glycolytic pathway (via GAPDH activity), pentose phosphate pathway (via G6PD activity), fermentation (via LDH activity), and the TCA cycle (via both IDH3 and SDH activity). Immediately following the enzymatic assay, the same (or serial) tissue sections are stained and analyzed using whole-tissue histocytometry. By combining these two methods, Haschemi and colleagues were able to resolve the metabolic activity of immune and non-immune cells *in situ* across multiple tissues and tissue environments, including human colon cancer. For instance, the authors observed that macrophages in the tumor microenvironment generally exhibited decreased metabolic activity across all enzymes assessed relative to tissue macrophages in healthy control tissue (192). This method comes with a few caveats. The first caveat relates to NBT itself. NBT is less sensitive to $O_{2}^{.–}$, more susceptible to reduction by tissue reductases, and more susceptible to autoxidation, than alternatives such as dihydroethidium, each of which can result in non-specific NBT fluorescence (193). Additionally, the enzymatic assays are performed at saturating concentrations of substrates and confactors, which may not faithfully reflect the metabolic activity of a cell in its native environment where substrates and cofactors are limiting. Finally, the activity of these enzymes is a measurement of steady state activity and does not necessarily reflect the flux of substrates nor the ultimate outcome of the intermediates and products formed. Nonetheless, this method is an important advance for measuring TRM metabolic activity *in situ*.

### Nuclear Magnetic Resonance, Single Photon Emission Computed Tomography, and Positron Emission Tomography

Molecular imaging of metabolism is not limited to light-based approaches. The molecular imaging umbrella includes three techniques that, while technically lacking in spatial resolution, make up for it with high specificity and minimal invasiveness: nuclear magnetic resonance (NMR), single photon emission computed tomography (SPECT), and positron emission tomography (PET). The use of each of these methods to study metabolism have been broadly reviewed in detail elsewhere (194–198). Both PET and SPECT rely on injection of radioactive tracers and detection of gamma ray emission, both allow for dynamic measurements, and both require registration to structural images (typically obtained using computed tomography), yet each has unique advantages and disadvantages (198). PET imaging generally has higher spatial resolution (4-6 mm for human scanners, 1 mm for small animal scanners), although advancements in micro-SPECT technologies have significantly improved this parameter for small animal imaging (10 mm for human scanners, below 1 mm for small animal scanners) (199). Additionally, the time required to acquire a full sequence of PET slices is significantly less than for SPECT. On the other hand, the tracers used for SPECT are significantly more stable than the tracers used for PET (200).

Both PET and SPECT have been used extensively to study macrophages *in vivo* in both murines and humans (201), owing largely to the advancements made in radiolabeled antibodies (known as radiopharmaceuticals) which allow for significantly enhanced cellular sensitivity and specificity (202). Examples of SPECT tracers that specifically target macrophages include imaging of a radiolabeled translocator protein (TSPO) small molecule in the inflamed ankles of a murine model of rheumatoid arthritis (203), of a second generation variant of the TSPO-targeting small molecule (iodo-DPA-713) in the pancreas, liver, and intestines of mice treated with cerulein (198), and of combined 14C-methionine and ^99m^Tc-methoxyisobutylisonitrile in a murine model of acute myocardial infarction (204). This latter study is an excellent example of the ability to simultaneously label cells and monitor metabolite uptake *in vivo* using micro-SPECT (205, 206). Examples of PET tracers that specifically target macrophages including imaging of combined ^89^Zr-Feraheme (FH) and ^18^F-Fludeoxyglucose (FDG) in non-human primates experiencing acute open wound injury or arthritis (207), of a long-circulating, dextran-coated nanoparticle labeled with 64Cu in atherosclerotic plaques of ApoE-deficient mice (208), and of phospholipid or apoA-I conjugated ^89^Zr-high-density lipoprotein (HDL) in an orthotopic mouse model of breast cancer (209). For both PET and SPECT, there are now numerous contrast agents and metabolites that can be imaged (210, 211). Given the significant advances already made, we believe future studies will be able to combine advances in macrophage-specific radiolabeling with unique metabolic tracers which will allow for *in situ* live animal imaging of TRM metabolism.

Although PET/SPECT allow for specific targeting of macrophages, these methods only allow one to measure uptake of a desired metabolite, not whether, how much, and in what way a given substrate is used. However, there are two alternative non-destructive/non-terminal approaches that are capable of measuring each of these parameters in living tissue, both of which rely on the principles of nuclear magnetic resonance (NMR): magnetic resonance imaging (MRI) and magnetic resonance spectroscopy (NMR). The use of these techniques to measure metabolism *in vivo* have been reviewed in great detail previously (194, 210, 212–215). Both MRI and MRS rely on the detection of energy exchange between the external magnetic field applied to the tissue/sample and atomic nuclei, however they vary in that MRI detects emitted radio frequency (RF) signal whereas MRS detects the chemical shift induced by the magnetic field interacting with electrons that surround the nucleus (“shielding”). Imaging of living tissue using ^1^H-MRS (proton MRS) has historically been challenging because of the high background signal that comes from water and the typically low abundance of desired metabolites combine to give low signal-to-noise ratio (SNR). However, several advances have led to improvements of MR-based *in vivo* imaging of cellular metabolism (194, 210, 212, 213). Specifically, magnets with increased field strengths, such as 11.7 T in humans and 21.1 T in rodents (216, 217), the use of body region-specific RF surface coils and MRI cryoprobes, and the use of infusion to introduce hyperpolarized metabolites [e.g., [6-^13^C, ^15^N_3_]-Arginine (218), for reviews, see (194, 210, 212, 213)], have each significantly improved the SNR for MR imaging of living tissue. Because of the ability to take repeated measurements of animals (or humans), hyperpolarized MR will continue to be an important method for studying TRM metabolism in health and disease.

### Mass Spectrometry-Based Methods

The workhorse for cellular metabolism broadly, and for macrophage biology specifically, is and will likely remain mass spectrometry (MS). The topic of ex vivo isolated tissue and cellular MS using gas chromatography (GC) or liquid chromatography (LC) have been reviewed in detail elsewhere. However we will highlight a series of excellent reviews on the broader technology, methodology, and approaches (from the Rabinowitz lab) (191, 219), the pioneering use of these techniques in mouse and human cancer (reviewed by the DeBerardinis lab) (220), and the application of these methodologies to study immunometabolism, particularly of T cells (reviewed by Jones and colleagues) (164). Together with the improved sensitivities of MS technology, we are excited by emerging accompanying approaches such as chemical isotope labeling nanoflow (nano)LC-MS, which potentially allows for LC-MS detection of thousands of metabolites in the ultralow cell number (e.g., 100-10,000) range (221). Unfortunately, each of the above MS-based methods requires isolation of cells or tissue, including potentially detrimental digestion and isolation steps. This is particularly problematic for TRMs, as noted above, because TRMs are integrally embedded in the tissue stromal meshwork which makes isolation difficult. Here, we briefly highlight three spatial MS imaging approaches that, if adopted and optimized, will allow for unparalleled investigation of *in situ* TRM metabolismml: MIBI-TOF, Nano-SIMS, and MALDI-MS imaging.

Both multiplexed ion beam-imaging by time of flight (MIBI-TOF) and nanoscale secondary ion mass spectrometry (Nano-SIMS) rely on detection of released secondary ions. For a review of SIMS principles, we recommend the following reviews (222, 223). Briefly, a surface (in this case the tissue section or tissue culture of cells) is rasterized with a focused primary ion beam which induces ejection of secondary ions. These secondary ions are then collected and analyzed for their mass/charge ratio using MS, typically TOF or sector field MS (for Nano-SIMS) but can also be analyzed using a quadrupole mass analyzer. In MIBI-TOF, a tissue of interest is prepared *via* standard histological approaches such as formalin-fixed, paraffin-embedded (FFPE) or fresh frozen (for immunofluorescence, and subsequently stained using antibodies conjugated to a unique metal isotope similar to those used for mass cytometry (CyTOF) (224). MIBI-TOF is capable of imaging at resolutions down to 260 nm (near-single molecule) with a modest field of view (800 μm x 800 μm). The detail that makes up the strength of MIBI-TOF is also its weakness. Specifically, MIBI-TOF requires antibodies that are capable of recognizing a protein of interest. Importantly, MIBI-TOF was recently used to study human cytotoxic T cells using a panel of metabolic pathway regulatory proteins such as transport proteins and essential enzymes (225). Thus far, MIBI-TOF has been restricted to proteins, although antibodies do exist against some metabolites, therefore it is theoretically possible to exploit these antibodies for MIBI-TOF in the future. On the other hand, Nano-SIMS improves on the successes of TOF-SIMS (226, 227) imaging by reducing the distance between the primary probe and the sample (228) which theoretically allows for subcellular (nanometer) detection of metabolites (222, 223). A beautiful example of the potential of Nano-SIMS was shown by i.v. injection and imaging of ^13^C-labeled triglyceride-rich lipoproteins or ^13^C-labeled fatty acids into mice (222). Nano-SIMS images of the cells in a tissue are generated either from independent analysis of single secondary ions (e.g., ^12^C^− 13^,C^−^) or the ratio of secondary ions (e.g., ^12^C^−^ to ^13^C^−^). Arguably the most important feature of Nano-SIMS is its ability to not only analyze metabolite pool sizes, but also analyze isotopic enrichment *in situ* at both relatively high lateral resolution and high spatial resolution. On the other hand, Nano-SIMS is limited by the number of unique masses that can be analyzed in parallel (up to 7 with the NanoSIMS 50L). One could imagine future TRM research combining NanoSIMS with other *in situ* methods such as spatial RNAseq or spatial MALDI imaging (see below) to study both cellular and subcellular TRM metabolism across large tissue areas.

**Figure 3:**
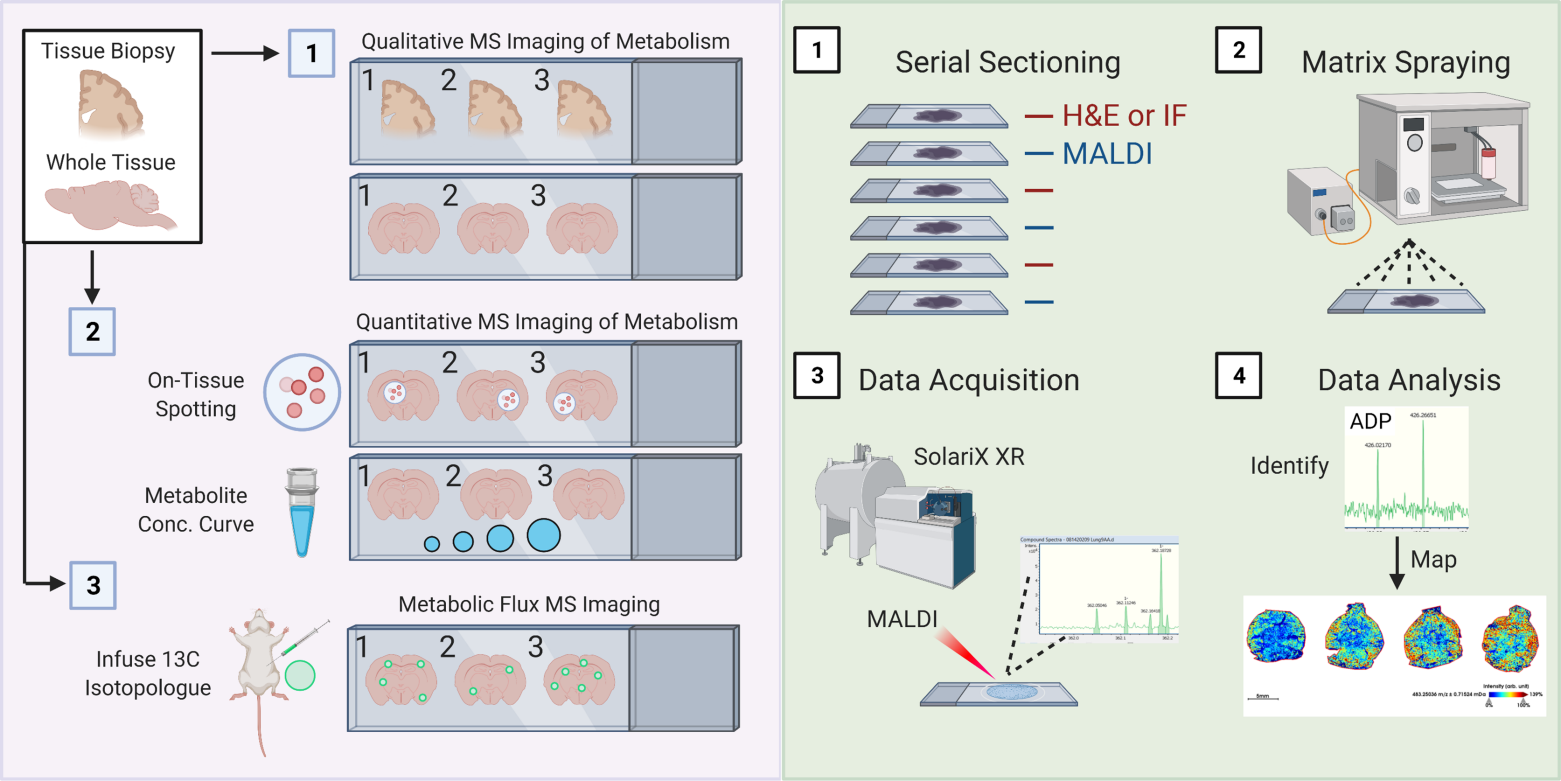
Schematic of spatial metabolomic analysis using MALDI Mass Spectrometry imaging. **(A)** 1. Isolated whole or sectioned tissue can be analyzed using a combination of untargeted approaches involving various MALDI matrix substances, such as 9-AA, allowing for profiling of a broad range of metabolite sizes and classes. 2. Quantitative analysis of metabolites of interest can be performed using two interconnected approaches: On-tissue spotting and metabolite concentration curves. On-tissue spotting involves taking a slurry of known metabolites (at known concentrations) and dabbing them onto tissues of interest. This allows for an internal reference standard to control for matrix deposition and laser excitation. Metabolite concentration curves allow for quantification of the concentration of a specific metabolite or finite number of metabolites of interest. 2. Metabolic flux analysis can be performed by adopting conventional *in vivo* infusion of ^13^C-labeled metabolites, such as uniformly-labeled ^13^C-Glucose into mice. **(B)** 1. Samples are cut entirely into serial sections. Interleaved sections are used for MALDI or H&E/immunofluorescence staining. 2. Slides used for MALDI are sprayed with matrix of interest using a standard MALDI matrix sprayer. Up to two slides can be sprayed and run at a given time, and slides with applied matrix should be imaged immediately. 3. Slides are then analyzed using a MALDI MSI (such as the SolariX XR MALDI-FT-ICR instrument, pictured). Slides are analyzed at a desired pixel resolution within the limits of the machine (e.g., 20-micron), meaning that the MALDI laser is rastered over the whole tissue surface, exciting 20-micron regions at a time. 4) Mass spectrometry peaks are subsequently analyzed for identity (see method) and mapped back onto H&E or IF stained slides.

Finally, it is important to imaging mass spectrometry (also known as mass spectrometry imaging, or MSI). Over the last several years, numerous techniques and equipment have been developed to perform MSI [reviewed here (229, 230)]. MSI, broadly speaking, is a collection of tools used to study the spatial distribution of molecules of interest (ranging from small metabolites up to more complex glycans, lipids, and proteins) across a region or the entirety of a tissue section. Here, we highlight matrix-assisted laser desorption/ionization (MALDI) MSI, including the recently released MALDI-2 (231), because of its improved sensitivity to small metabolites (including under 100 kDa) and improved spatial resolution (down to ~5-10-microns). Indeed, several modifications have allowed for dramatically improved mass accuracy, including the use of hybrid mass analyzers such as QTOF or Fourier-transform ion cyclotron resonance (FT-ICR) (232, 233). Much of this work has focused on spatial imaging of lipid metabolism down to 10-micron resolution (234, 235), however several proof-of-principle studies have demonstrated its utility to smaller metabolites including glutamine and glucose (236, 237). Importantly, MALDI MSI was also recently shown to be useful for isotope flux analysis *in situ* of ^13^C_5_-glutamine in the intestines (236). MALDI MSI can principally be used on tissue biopsies or whole tissues to perform qualitative MS of hundreds to thousands of metabolites, as well as perform quantitative and isotopic enrichment analyses of metabolites of interest (**Figure 3A**).

MALDI involves four key steps (**Figure 3B**). Tissues of interest must be immediately snap frozen, which are then cut onto appropriate slides using a cryostat (1). This step is essential to ensure minimal change in regional cellular distribution or tissue structure as well as to minimize changes in metabolites. Previous groups have also used FFPE tissues, which are fairly common in clinical settings, although report noticeable loss in abundance of desired metabolites. Principally, serial sections would be kept for immunofluorescence staining (and possibly also spatial RNAseq), which is necessary for subsequent cellular localization of identified metabolites. Then, a matrix material is applied to the tissue section using a matrix sprayer (such as the HTX TM-Sprayer) which helps to evenly distribute gaseous matrix substance onto tissues (2). Then, samples coated with matrix substance are rasterized with a laser at the desired pixel size which causes ablation and desorption of the matrix-coated sample (3). In the case of MALDI-2 (such as the one housed in the timsTOF Flex 2), a second laser has been added that allows for a 10x improvement in sensitivity and resolution (down to single cell) (231). Metabolites are ionized and accelerated for identification *via* MS, for example using FT-ICR (as illustrated in **Figure 3B**). Finally, data is first processed similar to typical MS studies, including identification of mass-charge ratios (m/z ratio, 4). However, it is worth mentioning that because of the incredible amount of data generated by scanning an entire tissue (or tissue section) coupled together with the need to register these m/z ratios to the individual pixels in an immunofluorescence image, it is likely that novel computational approaches will be required, especially as we begin to generate 3-dimensional renderings of whole tissues as illustrated. We anticipate registration tools, such as those long used for neuroimaging, may help in this endeavor.

## Conclusions

In this Review, we have highlighted exciting new findings on tissue-resident macrophage (TRM) metabolism. We define a macrophage’s time spent in a particular environment (or ‘niche’), and the features that define that environment, as its *habitare*. We believe *habitare* is apt, because it carries with it a quality that is more than just the cold residence in which one resides, but instead includes the richness and warmth that makes the place one resides in a home. We make the case that TRM metabolism is defined by their early entrance into this *habitare* and is informed over time by the factors that the TRM comes into contact with, both during homeostasis and inflammation. Clearly John Locke never imagined that one’s environment would imprint at the biological level, as it does for memory. We argue that TRMs are instructed in a similar way, first informed by intrinsic programs but ultimately, and continually, informed by their lived experience. Finally, we highlight the emerging technologies that we think will help move forward this important field of study. We believe that lowered costs, increased availability, and better analytical algorithms will allow for widespread adoption of these techniques, and we look forward to the future work to come.

## Author Contributions

GZ, PHVS, KRK and JSAP wrote, revised, and approved the manuscript for publication. All authors contributed to the article and approved the submitted version.

## Funding

This work was supported by NIH NCI 5R00CA237728-03 and the Parker Institute for Cancer Immunotherapy Career Development Award (Perry laboratory), NIH NCI 1R01CA248364-01A1 (Keshari laboratory), and MSKCC Cancer Center Support Grant P30CA008748.

## Conflict of Interest

The authors declare that the research was conducted in the absence of any commercial or financial relationships that could be construed as a potential conflict of interest.
